# P10 Rapid capture of uropathogenic bacteria and on-chip determination of antimicrobial resistance

**DOI:** 10.1093/jacamr/dlac004.009

**Published:** 2022-02-16

**Authors:** Andrea Dsouza, Rachel Hand, Chrystala Constantinidou, Theodoros N. Arvanitis, David M. Haddleton, Jérôme Charmet

**Affiliations:** Warwick Manufacturing Group, University of Warwick, Coventry, UK; Department of Chemistry, University of Warwick, Coventry, UK; Warwick Medical School, University of Warwick, Coventry, UK; Warwick Manufacturing Group, University of Warwick, Coventry, UK; Department of Chemistry, University of Warwick, Coventry, UK; Warwick Medical School, University of Warwick, Coventry, UK; Haute Ecole Arc Ingénierie, Neuchâtel, Switzerland

## Abstract

**Background:**

Urinary tract infection (UTI) is one of the most common bacterial infections responsible for increased annual incidence of antimicrobial resistance (AMR) cases. Clinical diagnosis of UTI AMR relies heavily on conventional urine culture and antibiotic susceptibility testing (AST) which has a turnaround time of ∼3 days. Often, irrespective of the infection status, antibiotics are prescribed to patients even before the test results are available, leading to non-judicious use of antibiotics. Over the years, several technologies have been developed for the rapid detection and diagnosis of UTI AMR, however, most of them are limited to traditional microbiological techniques and large laboratory equipment that are not readily available in low-to-middle income countries (LMICs). To address these diagnostic limitations, we are developing a rapid and affordable UTI-AMR diagnostic microfluidic device that is clinical friendly aimed at improving UTI management and AMR stewardship.

**Results:**

Our device enables the flow of a large volume of urine specimens for the capture/enrichment of uropathogenic bacteria and determination of AST via a porous membrane that is augmented with a multifunctional polymer-based material. Important objectives for the development of UTI AMR diagnostic microfluidic device are: (i) development of a multifunctional polymer-based material; and (ii) validation of UTI AMR diagnostic device. We have successfully developed a polysaccharide-based platform to (i) selectively capture uropathogenic bacteria from urine specimen by immobilizing concanavalin A (con A) lectin as bacterial capture agent on the polymer surface via chemical modification; (ii) encapsulate and release bacterial nutrient media and antibiotics for AST; and (iii) detect AST via encapsulation of bacterial growth indicator. In addition, we have also determined the development of methacrylate-based and acrylamide-based synthetic polymer-based material for our application. Further, we have demonstrated the uniform augmentation of the polysaccharide-based polymer onto porous membrane via dip-coating technique for on-chip bacterial capture/enrichment and AST in fluid (urine) flow conditions. The porous membrane is a conducting material which enables us to perform electrochemical measurements such as impedance spectroscopy that accelerates the detection process of antibiotic susceptibility. As a proof-of-concept, we have determined the capture of biosafety level I *Escherichia coli* expressing kanamycin resistance gene on chemically surface modified polysaccharide-based polymer containing con A and the antibiotic susceptibility of captured bacteria against different antibiotics with and without the porous membrane. We have quantitatively determined the limit of detection of *E. coli* on multifunctional polysaccharide-based polymer material.

**Conclusions:**

The utility of the UTI AMR microfluidic device in clinical settings enables clinicians to make informed decisions on the most appropriate antibiotic for treatment in less than a day. Integration of impedance spectroscopy will further accelerate the detection by significantly reducing the time of detection. Further, the device allows for off-chip analysis by retrieving the captured uropathogenic bacteria to perform high throughput sequencing for identifying AMR genetic determinants. Therefore, with the ability to selectively capture uropathogenic bacteria and determine AST in a short time, our technology has the potential to overcome some of the current limitations in UTI AMR diagnostics.

